# *QuickStats*: Age-Adjusted Death Rates* of Heart Disease and Cancer, by Sex — United States, 2010–2020

**DOI:** 10.15585/mmwr.mm7115a4

**Published:** 2022-04-15

**Figure:**
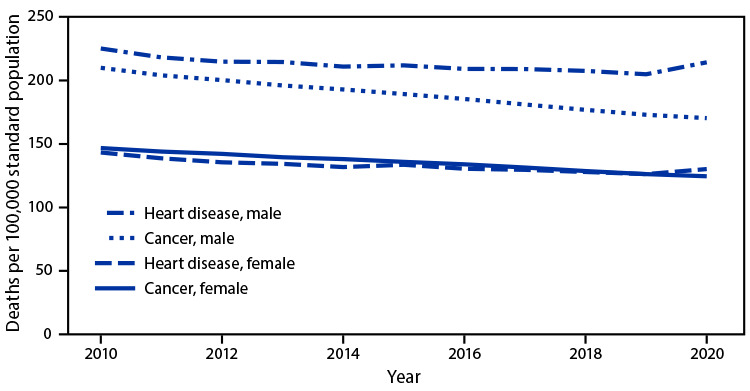
Age-adjusted cancer and heart disease death rates for both males and females declined steadily from 2010 to 2019. Cancer death rates continued to decline for both males and females during 2019–2020 to 170.3 per 100,000 population (males) and 124.5 (females) in 2020. The pattern was different for deaths caused by heart disease for both males and females. Heart disease death rates increased during 2019–2020 to 214.2 (males) and 130.2 (females) in 2020. During 2010–2020, higher death rates were reported for males than females for both heart disease and cancer, with the cancer death rate for males exceeding the heart disease death rate for females.

**Source**: National Center for Health Statistics, National Vital Statistics System, Mortality Data. https://www.cdc.gov/nchs/nvss/deaths.htm

